# Selection on synonymous codons in mammalian rhodopsins: a possible role in optimizing translational processes

**DOI:** 10.1186/1471-2148-14-96

**Published:** 2014-05-03

**Authors:** Jingjing Du, Sarah Z Dungan, Amir Sabouhanian, Belinda SW Chang

**Affiliations:** 1 Department of Ecology & Evolutionary Biology, University of Toronto, 25 Harbord Street, Toronto, ON M5S 3G5, Canada; 2 Department of Cell & Systems Biology, University of Toronto, 25 Harbord Street, Toronto, ON M5S 3G5, Canada; 3 Centre for the Analysis of Genome Evolution and Function, 25 Harbord Street, Toronto, ON M5S 3G5, Canada

**Keywords:** Mutation-selection model, dN/dS, Codon-based likelihood models, Visual pigment evolution

## Abstract

**Background:**

Synonymous codon usage can affect many cellular processes, particularly those associated with translation such as polypeptide elongation and folding, mRNA degradation/stability, and splicing. Highly expressed genes are thought to experience stronger selection pressures on synonymous codons. This should result in codon usage bias even in species with relatively low effective population sizes, like mammals, where synonymous site selection is thought to be weak. Here we use phylogenetic codon-based likelihood models to explore patterns of codon usage bias in a dataset of 18 mammalian rhodopsin sequences, the protein mediating the first step in vision in the eye, and one of the most highly expressed genes in vertebrates. We use these patterns to infer selection pressures on key translational mechanisms including polypeptide elongation, protein folding, mRNA stability, and splicing.

**Results:**

Overall, patterns of selection in mammalian rhodopsin appear to be correlated with post-transcriptional and translational processes. We found significant evidence for selection at synonymous sites using phylogenetic mutation-selection likelihood models, with C-ending codons found to have the highest relative fitness, and to be significantly more abundant at conserved sites. In general, these codons corresponded with the most abundant tRNAs in mammals. We found significant differences in codon usage bias between rhodopsin loops versus helices, though there was no significant difference in mean synonymous substitution rate between these motifs. We also found a significantly higher proportion of GC-ending codons at paired sites in rhodopsin mRNA secondary structure, and significantly lower synonymous mutation rates in putative exonic splicing enhancer (ESE) regions than in non-ESE regions.

**Conclusions:**

By focusing on a single highly expressed gene we both distinguish synonymous codon selection from mutational effects and analytically explore underlying functional mechanisms. Our results suggest that codon bias in mammalian rhodopsin arises from selection to optimally balance high overall translational speed, accuracy, and proper protein folding, especially in structurally complicated regions. Selection at synonymous sites may also be contributing to mRNA stability and splicing efficiency at exonic-splicing-enhancer (ESE) regions. Our results highlight the importance of investigating highly expressed genes in a broader phylogenetic context in order to better understand the evolution of synonymous substitutions.

## Background

Selection is well-known to drive non-synonymous substitutions because such mutations alter the amino acid sequence, and thus the biochemical nature, of proteins [1]. Though less intuitive, selection can also affect synonymous substitutions, manifesting as codon usage bias (the non-random use of synonymous codons) in a wide variety of organisms [2-5]. Codon usage bias can result from both natural selection and mutational bias, with the relative influence of each varying across species (for review see [4-6]). Mutational bias arises from biochemical mechanisms that lead to certain bases changing more than others (e.g. transcription-associated [7,8]). By contrast, selection is thought to be the main driving force behind codon usage bias in fast-growing organisms with large population sizes (e.g. *E. coli* and yeast, [8-12]). In mammalian genomes, however, natural selection is considered to exert a minor, or even undetectable, effect on codon usage [4,5,13,14]. This is because the small effective population sizes (N_e_ < 10^6^) of most mammal species mean that the effect of genetic drift is likely to overwhelm the small selection coefficients that distinguish most synonymous codons (1/(2N_e_) > s) [4,15]. Genes with extremely high expression may provide exceptions to this rule, however, and have been associated with strong codon usage bias in non-mammalian species due to an increased selection pressure to minimize errors in gene expression [16]. Essentially, the redundancy of the genetic code allows the efficiency of gene expression to be tuned by selective forces [17]. This is thought to lead to fixation even when effective population sizes are relatively modest [4].

Evidence for selection on synonymous codons can be statistically evaluated with computational models. Base composition, codon frequencies, and substitution rates at synonymous sites can deviate from the expectations of neutral evolution, implicating selection [18-26]. However, classic phylogenetic codon models assume that the synonymous substitution rate (dS) is constant among sites (not affected by selection, [27]), and that the rate variation among codons is solely due to the variation at non-synonymous sites (dN) [28,29]. Of course, this assumption is not necessarily true for all genes [6]. Several new models relax this constraint by estimating dN and dS separately from discrete distributions of *n* categories (*n* > =3) [30], or by using a gamma distribution [31]. Population genetic studies have used alternate modeling frameworks, differing from the phylogenetic codon models in that the usage of synonymous codons is the product of interactions among mutational bias, natural selection and genetic drift [23-26]. By incorporating population genetics ideas into a phylogenetic likelihood framework, Yang and Nielsen [32] developed a full codon substitution model for synonymous sites, and provided a test to directly determine whether selection is acting on synonymous substitutions in a phylogenetic context. Their model incorporates two separate parameters to account for the effects of mutational bias and selection. Given a null model that only assumes the effect of mutational bias, a likelihood ratio test can determine whether codon usage patterns are due to mutational bias alone. These models are particularly useful because they not only allow for a direct test of selection on synonymous codons, but also allow the selective strength on each codon to be quantified.

Synonymous codon selection seems primarily influenced by post-transcriptional and translational pressures [5,14,33], which result from the interaction of several mechanisms. These include: selection for translational accuracy, proper protein folding, mRNA stability, and more efficient splicing control. All of these selective mechanisms can leave distinguishable signatures in protein coding sequences. For example, proper protein folding during translation can be dependent on both translational accuracy (correct incorporation of amino acids) and controlling the elongation rate in structurally sensitive regions (reviewed in [34] and [17]). Strategic control of the elongation rate and translational pausing can be achieved with codon usage bias, and a number of studies have demonstrated correlations between codon usage patterns and protein secondary structure in multiple species [35-42]. This is because tRNAs have varying concentrations inside the cell, and rare tRNAs are less quickly recognized by the ribosomes due to their lower abundance [43]. Codon bias can also be influenced by selection for mRNA stability. In humans and mice, optimal codons for translation are mostly GC-ending [44,45]; these codons are thought to decrease both mRNA degradation rates *in vitro*[46] and the Gibbs free energy of mRNA secondary structure [47,48]. Lastly, selective constraint for splicing control also seems to cause low synonymous substitution rates in splicing associated regions, such as purine-rich exonic splicing enhancers (ESEs) [49] and exon-intron junctions [50,51].

Despite the mechanistic evidence for codon usage bias, and the known association between codon usage bias and high gene expression, the majority of studies investigating selection on synonymous codons in mammals have focused on genome-wide patterns and have sampled only a limited diversity of mammal species (for review see [5,6]). If there is potent selection on synonymous codons in mammals, then signals of selection are most likely to be detected in genes with extremely high expression. The most highly expressed genes in mammals include members of the G protein-coupled receptor (GPCR) family [52], and some of the most well understood GPCRs are the visual pigment opsins. Opsins are the subject of numerous molecular evolutionary studies [53]. In particular, rhodopsin, a seven-transmembrane GPCR [54] that mediates dim-light vision in vertebrates [55], may be a good model system for studying selection on synonymous sites. Rhodopsin has a density of 25000 μm^−2^ in mammalian rod photoreceptor cells, with approximately 7 × 10^7^ proteins per rod outer segment, making it one of the most highly expressed proteins in the mammalian genome [56]. There is also a wealth of existing sequence and functional data for this protein from many species, its crystal structure is established [57], and its well-understood involvement in the visual pathway [54] can provide clear links between patterns of selection and organismal biology. In this study, we combine statistical approaches for detecting synonymous selection with investigations of codon usage bias in order to infer selection pressures acting on specific translational mechanisms. Focusing on a single highly expressed gene, mammalian rhodopsin, allows us to both distinguish synonymous codon selection from mutational effects and to analytically explore the underlying functional mechanisms (translational accuracy, protein folding, mRNA stability, splicing control) at work.

## Methods

### Estimating codon usage bias

The rhodopsin coding sequences were downloaded from the NCBI GenBank database using keywords and BLAST with a python script. The echidna rhodopsin sequence was provided by Bickelmann et al. [58]. Eighteen rhodopsin sequences were chosen to represent a diversity of mammals from most major taxonomic groupings. Accession numbers and sequence lengths for all the sequences used are given in Additional file 1: Table A1. Rhodopsin intron sequences were also available for eleven species on the NCBI and Ensemble databases, so we used them as a comparison dataset (Additional file 1: Tables A1 and A2). Sequences were aligned using the codon model in the PRANK Probabilistic Alignment Kit [59]. The phylogeny used in this study was based on established relationships among species [60-63] (Additional file 2: Figure A1).

Codon usage bias was measured using the Relative Synonymous Codon Usage (RSCU) values calculated in the program GCUA1.0 (General Codon Usage Analysis, [64]). Each of the sixty-one universal genetic codons has one RSCU value, which is used to quantify the observed abundance of a codon relative to the expected number given equal usage of alternative codons for each amino acid. A high RSCU value means that a codon has high abundance and therefore high usage bias. Heat maps of RSCU values were constructed using CIMMiner [65].

### Investigating selective constraint on synonymous substitutions

To investigate the synonymous substitution rates across sites in rhodopsin, we implemented the Dual model in HyPhy 2.2 [66]. In this model, dN and dS are estimated separately within discrete distributions of *n* equally probable classes (*n* = 3 in our study) [30]. A likelihood calculation is then used to compute the empirical Bayes posterior dS at each site [30] (Additional file 3: Figure A2). The non-synonymous model in HyPhy is the null condition for the Dual model and assumes variable dN but constant dS across sites. A Likelihood ratio test (LRT) comparing the Dual model to the non-synonymous model (degrees of freedom = 4) was constructed to test the null hypothesis that dS is not variable across sites.

To statistically test whether selection was acting on synonymous sites of mammalian rhodopsins, the mutation-selection models of Yang and Nielsen [32] were implemented in the CODEML program of PAML4.7 [67]. These models build on two separate parameters for a newly arisen mutant allele: the probability of mutation (effect of mutational bias or mutating tendency towards the mutated nucleotide) and the probability of fixation (effect of selection coefficients). The fixation probability of a newly arisen mutant is determined by its fitness change (selection coefficients) and effective population size, which are concepts adapted from population genetics [68-70]. Relative codon fitness is computed by comparing the selection coefficient of each codon to an arbitrary codon (the model uses GGG); positive or negative values indicate that the codon is respectively more or less advantageous than GGG. An LRT compares the null model (FMutSel0) to the alternative model (FMutSel); the instantaneous synonymous substitution rate is considered to be proportional to the parameter of mutational bias in the FMutSel0 model, and both mutational bias and selection in the FMutSel model. Thus, the test directly evaluates whether selection is acting on synonymous substitutions. The test statistic is twice the difference in maximum likelihood values between nested models, and significance is calculated using a *χ*^2^ distribution with the appropriate degrees of freedom (the difference in the numbers of parameters between two models, df = 41 in this case). In our study, the estimated values of codon fitness were used to reveal selectively preferred synonymous codons in rhodopsin, which we defined as having the highest fitness among all synonymous codons for each amino acid.

In addition to modeling the evolution of synonymous substitutions, the mutation-selection models also estimate ω (dN/dS) for modeling the evolution of non-synonymous substitutions [32]. So far, the FMutSel/FMutSel0 model pair is only incorporated with the M0 and M3 models in PAML4. Model M0 assumes constant ω among branches and sites, whereas M3 allows ω to vary across sites according to a random distribution with n discrete categories (n = 2 in this study). We therefore carried out four analyses and two LRTs: an M0 set (FMutSel-M0, FMutSel0-M0), and an M3 set (FMutSel-M3, FMutSel0-M3). Estimated parameters of mutational bias and selection coefficients between the FMutSel-M0 and the FMutSel-M3 model were compared to check the consistency of the likelihood estimation. Analyses were run three times with different initial ω values (0.01, 1, 10) to capture local optimization.

### Tests for translational efficiency, mRNA stability, and splicing

To test for selection on translational accuracy (correct incorporation of amino acids in the polypeptide chain), we determined the correlation between C-ending codons, which are known to be favoured in human and mouse translational selection [44,45] (these also had the highest fitness in our mutation-selection models), and conserved amino acid positions using the Mantel-Haenszel test. Akashi [71] used the test to investigate codon usage bias and translational accuracy in *Drosophila*. Codons were divided into two groups: preferred and un-preferred (as indicated by a significant increase in relative synonymous codon usage between the least and the most highly expressed genes), and site positions were designated as either conserved or non-conserved. This set-up effectively allows the correlation between preferred codons and conserved amino acids positions to be tested. A significantly high correlation would suggest that selection is acting on preferred codons to increase translational accuracy [45,72]. As such, we replicated the set-up of Akashi [71] and defined the first factor by designating four-fold synonymous codons as either ending or not ending with C, which we found to have the highest fitness values according to the MutSel models in all cases except for leucine. We defined conserved sites as those with the same amino acids for all the rhodopsin genes in our dataset.

Because rhodopsin is a transmembrane protein that requires membrane integration while being translated and folded [73], we expected that loops and helices might differ in their codon usage bias in correlation with relative tRNA abundances given that these motifs are known to vary in their sensitivity to folding errors [18,25]. We used tRNA copy numbers as a proxy for the abundance of tRNA species in the cell, and then used these relative abundances to categorize four-fold synonymous codons as having either “fast” or “slow” translation rates (corresponding to high or low abundance of tRNA matches respectively, assuming C- and T-ending codons are recognized by the same tRNAs, Additional file 1: Table A3). We compared the proportion of fast and slow codons in loops vs. helices using a Mantel-Haenszel test. Other studies have found a positive correlation between cellular tRNA and tRNA gene copy number in a variety of species including *E. coli.*[74]*, S. cerevisiae*[75], *C. elegans*[76], and human [44]. Data for tRNA gene copy numbers were obtained from the Genomic tRNA Database (http://lowelab.ucsc.edu/GtRNAdb/) [77], which is based on the tRNAscan-SE analysis of complete genomes [78]. Thirteen out of the 18 species in our dataset had available annotations of tRNA genes (all species except for the echidna, dunnart, polar bear, manatee, and galago). We also compared the rate of synonymous substitutions at individual sites between helices and loops using a Mann–Whitney U test, and the variation in dS between helices and loops using Levene’s test. The predictions of helix and loop regions were based on the bovine rhodopsin 3D structure [57], which is commonly used as a model to study mammalian rhodopsins.

For testing selection on mRNA stability, we determined the correlation between GC-ending codons, which are thought to decrease mRNA degradation rates [46] and result in more energetically stable secondary structures [47,48], and pairing site positions in the rhodopsin mRNA 2D structure. As such, we applied the Mantel-Haenszel test again, this time designating four-fold synonymous codons as those either ending or not ending with GC, and classifying site positions as either paired or non-paired in the mRNA secondary structure. Increased base-pairing in mRNA structure is thought to increase mRNA stability, so selection may be acting on sites that form stems (paired sites) in mRNA secondary structures [47,48]; we used computational algorithms to determine these sites in rhodopsin. The primary computational approach to predict RNA secondary structure is the Minimum Free Energy (MFE) algorithm, which estimates the thermodynamic parameters of each possible structural mRNA permutation and chooses the one with minimum free energy (most negative value) [79]. Another algorithm also determines the Centroid structure (the permutation with the minimum base-pair distance to all others in the thermodynamic ensemble) as a comparison to the MFE structure. A reliable prediction is indicated if the MFE and Centroid structures are highly similar. These methods assume that a given sequence will fold into the structure that is thermodynamically most efficient [80]. We implemented these algorithms in the RNAfold server of the University of Vienna RNA website (http://rna.tbi.univie.ac.at/) [81-83]. All analyses were performed under the default settings of the server. The paired and non-paired sites were identified under the optimal mRNA 2D structure predicted by both algorithms.

Finally, we also investigated the role of selection on splicing site recognition. In the gene splicing process, three necessary motifs are involved: a 5’ splice site (5’ss), a branch point, and a 3’ splice site (3’ss) [84]. However, this tripartite signal is often not sufficient for intron excision [85]. The mRNA sequence or structure in the vicinity of the 5’ss and 3’ss motifs is also known to play an important role in splice site recognition [86]. Exonic splicing enhancer (ESE) sequences, which enhance splicing at nearby sites [49,87], are an important component in this context. If selection is acting to control efficient splicing, it should prevent synonymous mutations that might disrupt the splicing-associated motifs in exons, such as ESEs. Therefore, we investigated selection for efficient splicing control by examining whether the ESE regions show slower synonymous substitution rates than non-ESE regions.

Mammalian ESEs were identified initially as purine-rich sequences that are associated with specific SR-family proteins [88]. There has been no study identifying ESEs in rhodopsin so far, so putative ESE hexamers were predicted using the RESCUE-ESE (Relative Enhancer and Silencer Classification by Unanimous Enrichment) web server (http://genes.mit.edu/burgelab/rescue-ese/) [89]. This tool summarizes the results of a computational study of the human genome and its subsequent experimental validation. In RESCUE-ESE, human and mouse are the only two mammalian species in our dataset whose putative ESE hexamers have been predicted [89,90]. As such, only putative rhodopsin ESEs for human and mouse were obtained using our sequences to search for matching motifs in the ESE database. We compared the dS among sites in putative ESE regions identified in both human and mouse to the dS of non-ESE boundary sites using a Mann–Whitney U test. Boundary sites were defined as sites that are non-ESE in both species, and fall within five amino acids upstream of a shared 5’ or downstream of a shared 3’ ESE site.

## Results

In this study, we implemented a series of computational methods to test for selection, and to investigate support for the various possible selective mechanisms acting on synonymous sites in mammalian rhodopsins. We collected a dataset of both exons and introns, sampling broadly across mammals (18 mammals, 11 of them with available intron data). In summary, there was evidence for selection on synonymous sites, and a greater codon-usage bias towards C-ending codons in conserved amino acid positions. We also found that GC-ending codon bias likely contributes to mRNA secondary structure stability, and that significantly lower dS in ESE than non-ESE regions indicates selection pressures are conserving important splicing sites. Finally, codon bias may also facilitate proper protein folding by mediating the translation elongation rate in helix and loop domains.

**Figure 1:**
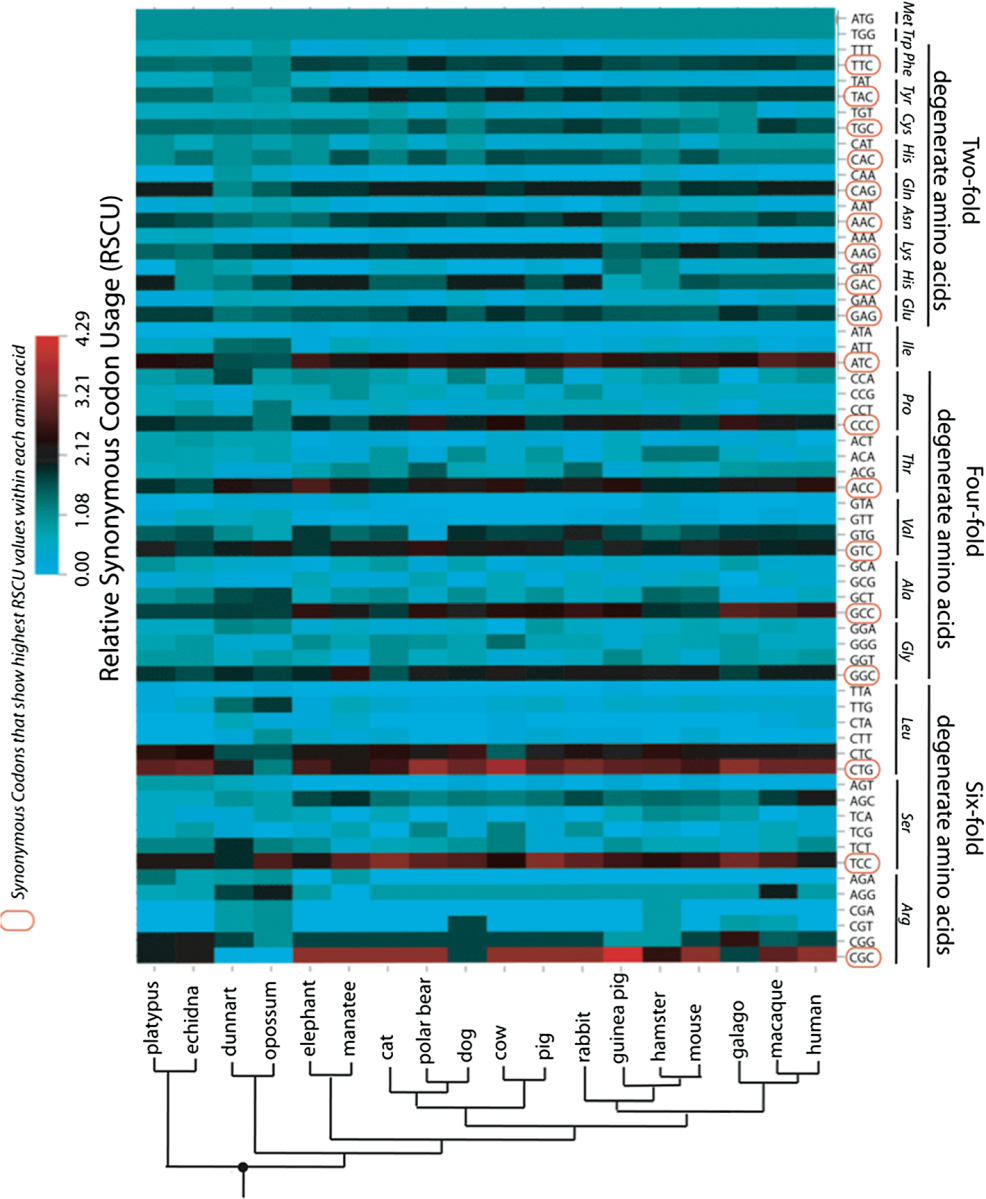
**Heat map of RSCU values for mammalian rhodopsin sequences.** Each column represents a species and each row represents a codon, with the corresponding amino acid abbreviations. The higher the RSCU value, the more abundant the codon is in the sequence. Codons with the highest RSCU values per amino acid are highlighted with a red background. C-ending codons in all the amino acids except for leucine show the highest RSCU values.

Before proceeding with models that explicitly test for the presence of selection on synonymous codons, we first tested for variability in synonymous substitution rates (the null condition being that all sites have comparable rates, with none more conserved or more diversified than others). We found significantly variable substitution rates across synonymous codon sites; the likelihood ratio test comparing the Dual model (allowing dS to vary across sites, [30]) to the Non-synonymous model (assuming constant dS across sites) in HyPhy2.2 [66] was significant (LRT p-value < 10^−5^, df = 4). According to the relative synonymous codon usage (RSCU) values, C-ending codons were the most abundant in almost all the codon families (Figure 1, Additional file 1: Table A4). We only investigated four-fold degenerate codons and the four-fold portion of six-fold degenerate codons so that all four bases could be represented at 3rd synonymous codon positions (for number of four-fold degenerate sites see Additional file 1: Table A1). We also found that the mean percentage of C nucleotides at four-fold degenerate sites (Additional file 1: Table A2) was significantly higher than the C content in introns, suggesting that mutational bias is not driving the observed variation in synonymous codon usage (Paired t-test: mean ± SD; 50.9 ± 3.9 vs. 26.0 ± 3.4; df = 10; p-value < 0.001).

To directly test whether synonymous sites of mammalian rhodopsins are under selection, we analyzed the coding sequences of our rhodopsin dataset using the mutation-selection models [32] in PAML 4.7 [67]. Four models within two sets were applied: an M0 set (FMutSel-M0, FMutSel0-M0) and an M3 set (FMutSel-M3, FMutSel0-M3). The LRTs comparing the FMutSel to FMutSel0 model were significant in both the M0 and M3 sets (p-value < 0.001, Table 1). These results suggest that there is significant selective constraint on synonymous substitutions of rhodopsin sequences across mammals.

**Table 1:** Parameter estimates and LRTs in the mutation-selection models

**Model**	**np**	**lnl**	***p-*** **value of LRT**	**к**	**ω**	**π**_C_^*^	**π**_G_^*^	**π**_T_^*^	**π**_A_^*^
**M0-set (alternative FMutSel-M0 tested against null FMutSel0-M0)**									
FMutSel0-M0	40	−6015.3	*N/A*	3.22	0.075	0.45	0.29	0.13	0.13
FMutSel-M0	81	−5878.3	**3.14 × 10**^**−36**^**(df = 41)**	2.94	0.050	0.19	0.20	0.20	0.42
**M3-set (alternative FMutSel-M3 tested against null FMutSel0-M3**									
FMutSel0-M3	42	−5860.4	*N/A*	3.28	ω_0_ = 0.012 ω_1_ = 0.431 p_0_ = 83.0%, p_1_ = 17.0%	0.45	0.29	0.13	0.13
FMutSel-M3	83	−5722.8	**1.99 × 10**^**−36**^**(df = 41)**	3.03	ω_0_ = 0.006, ω_1_ = 0.272, p_0_ = 81.6%, p_1_ = 18.4%	0.19	0.20	0.18	0.43

np is the number of parameters in the model, lnl is the log likelihood score, *p*-value is the result of likelihood ratio tests (LRTs), df is the degrees of freedom in LRTs, к is the transition/transversion ratio, ω is the non-synonymous/synonymous substitution ratio, π_N_^*^ (N = C, G, T, A) is the parameter of mutational bias for C, G, T, A, respectively.

**Figure 2:**
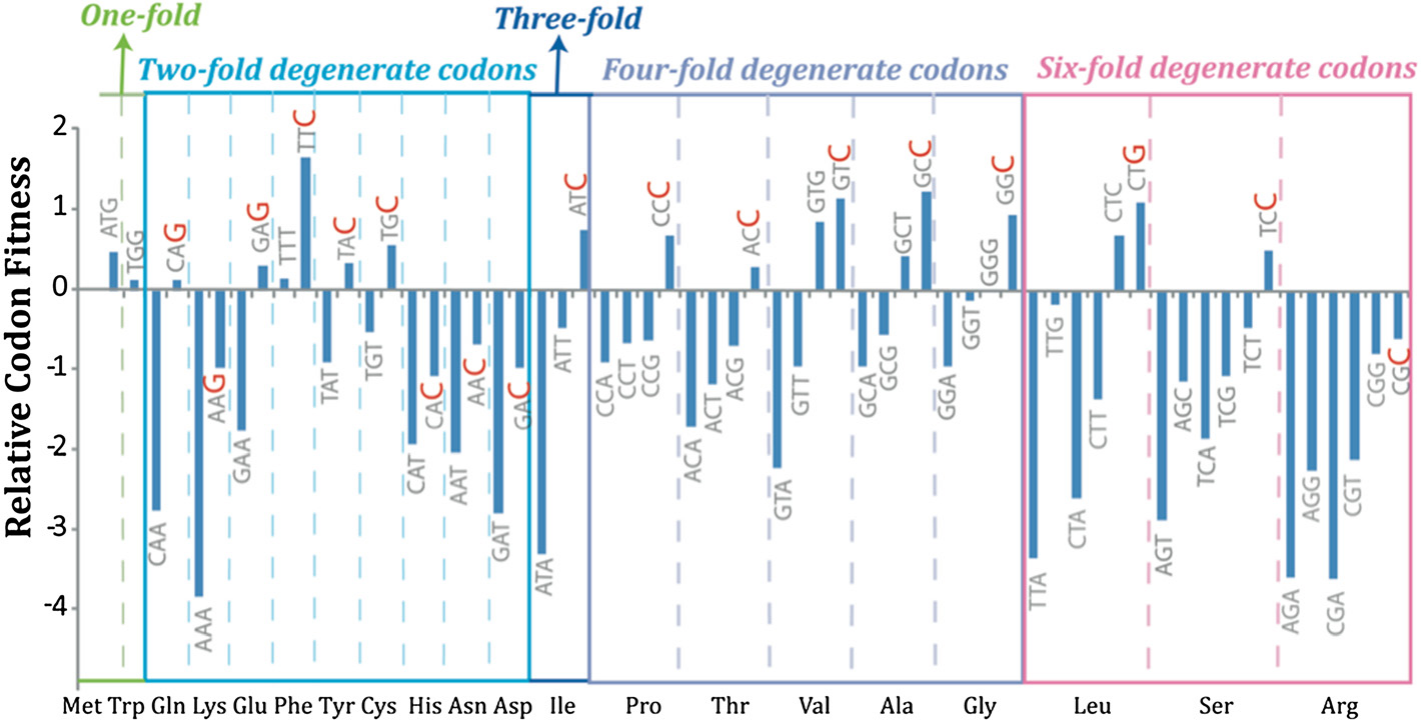
**Relative fitness distribution for mammalian rhodopsin codons.** The codons are grouped by the degeneracy of the coded amino acid, and the associated amino acids are marked at the bottom line of the plot. The fitness values are estimated in the mutation-selection model, M0-FMutSel [32]. The 3rd nucleotide of codons that have the highest fitness in each amino acid are highlighted in red.

After the role of selection on synonymous substitutions was confirmed, we determined which synonymous codons were selectively preferred in our dataset. Almost all of the four types of degenerate amino acids showed a consistent trend where, among codon families with C-ending degenerates, codons ending with C had the highest fitness. The only exception was leucine, for which the G-ending codon had highest fitness (Figure 2). Furthermore, a comparison of the frequency of C-ending codons at conserved and non-conserved amino acid sites revealed a statistically significant association between C4 codon (four-fold codons ending with C) usage and amino acid conservation (Mantel-Haenszel test: odds ratio = 1.4; p-value = 0.0004). This indicates that C-ending codons are more abundant at conserved amino acid positions, a pattern that may have significance for translation, given that these codons generally corresponded to the most abundant tRNAs (Additional file 1: Tables A3 and A4).

To investigate the potential effects of protein secondary structure on synonymous site selection we compared codon frequencies between rhodopsin loops and helices. We used tRNA gene copy numbers to assign relative translation rates to four-fold synonymous codons; either “fast” or “slow” depending on whether codons were translated by tRNAs with the highest or lowest copy numbers respectively. We found that slowly translated codons constitute 31% of synonymous codons in loops, compared to 23% in transmembrane helices, a difference that was significant (Mantel-Haenszel test, odds ratio = 1.6, p-value = 0.008). We also compared the site-specific dS between rhodopsin loops and helices, but the difference was not significant (Mann–Whitney U test: median = 1.01 at loop sites vs. 1.00 at helix sites; p-value = 0.893). However, we thought there might be differences in average dS depending on location in the tertiary structure. In fact, the *variance* in mean dS among loops was significantly higher than among transmembrane helices (Levene’s Test: mean ± SD; 0.964 ± 0.123 vs. 1.000 ± 0.032; p-value = 0.022). We found that dS was on average lowest in the first two loops (0.832 and 0.811) and generally increased in each loop towards the last, which had the highest average dS (1.122).

The bias we found towards C-ending codons in conserved regions might be associated with mRNA stability as well. There were a significantly higher proportion of GC-ending codons at paired sites than at non-paired sites in mRNA 2D structures (Mantel-Haenszel test, odds ratio = 2.2; p-value = 4.8 × 10^−17^). This suggests selective constraint acts on GC-ending codons to maintain mRNA stability, which is consistent with previous studies showing the stabilizing effects of GC-ending codons on mRNA structure [46-48]. Moreover, because our results showed that C was more abundant overall, we sought to determine whether C was more important than G for maintaining mRNA secondary structure in our dataset. We exchanged the GC content at four-fold degenerate sites (i.e. replaced C nucleotides with G and vice versa) to keep the numbers of paired sites in the secondary structures consistent, with the expectation that a less stable mRNA structure would result. The minimum free energy algorithm and thermodynamic ensemble predictions were both used to calculate the free energy of the mRNA secondary structures (see Methods for details). However, we found that GC-swapped sequences had lower predicted free energy than the original sequences (Additional file 1: Table A5), suggesting that G-ending codons contribute more to mRNA stability than C-ending codons.

Finally, to determine whether selection at synonymous sites was influencing the splicing process, we compared the synonymous substitutions rates of putative exonic splicing enhancer (ESE) regions to those of non-ESE regions in human and mouse rhodopsin (in our dataset, only human and mouse currently have genome-wide predicted putative ESE hexamers). The 5’splicing sites (GT) and 3’splicing sites (AG) were conserved among mammalian rhodopsins (except one site in dog and one site in cat, intron data not shown), suggesting the presence of selection on splicing control for introns. Sites that were in putative ESE regions of both human and mouse rhodopsin also had lower synonymous substitution rates on average compared to non-ESE boundary sites, further confirming the presence of selection in ESE regions (Mann–Whitney U test: median = 0.99 at ESE sites vs. 1.06 at non-ESE boundary sites; p-value = 0.039).

## Discussion

In this study, we investigated the strength and the underlying mechanisms of selective constraint on synonymous codons in the highly expressed mammalian rhodopsin gene [56]. We found significantly variable rates of synonymous substitution (dS), and significant evidence that there is selective constraint acting on synonymous sites. These patterns likely result from a high selective preference for C-ending codons throughout the rhodopsin coding sequence, a bias that appears to influence translation, mRNA stability, and splicing. We thus present a comprehensive study of selection at synonymous sites in mammalian rhodopsin incorporating both substitution rate modeling, and mechanistic lines of evidence for selection pressures related to translational processes.

Given that selection on synonymous sites in mammals is generally assumed to have a minor effect on codon usage bias [4,5,13,14], our study demonstrates that this may not be true for highly expressed genes. In non-mammalian species, highly expressed genes are characterized by strong codon usage bias because of greater selection pressure for both fast and accurate translation (e.g. [43,91-93]), yet little attention has been given specifically to highly expressed mammalian genes. Because rhodopsin has very high expression levels in mammals [56], the gene should be experiencing considerable selection pressure to minimize translation errors while maintaining a high translation rate. Previously documented biases in mammalian rhodopsins towards G- and C-ending codons have already hinted at synonymous site selection [94], but our study focuses exclusively on this highly expressed gene in a phylogenetic context, a setup that affords us the liberty to also investigate mechanisms of selection.

### Selection to optimize translation and protein folding

We found evidence that synonymous codon selection in mammalian rhodopsin may influence translation accuracy as shown by a higher abundance of C-ending codons in conserved sites. Specifically, for four-fold codons, tRNAs with A in the first anti-codon position (A_34_ in the tRNA sequence) were generally the most abundant, and these get converted to inosine (I) in eukaryotes [95]. The most abundant four-fold codons in our dataset were C-ending, which match preferentially to these tRNAs [96]. This suggests that rhodopsin may be experiencing a general selection pressure to decrease amino acid misincorporation errors (especially in conserved regions where protein function can be compromised) while maintaining a high overall translation rate [93]. Although a C-I interaction does not have as high affinity as a C-G interaction, the pairing is considerably more favorable than other wobble pairs [96]. Even though C-ending codons have some chance of being deaminated to U, they will still be recognized by inosine-converted tRNAs [96]. Alternately ending codons may be even less optimal. For example, C_34_ to U_34_ deamination on tRNAs can make G-ending codons more error prone because of the less favorable geometry of G-U pairings, and because U_34_ tRNAs can pair with codons ending in other bases [97].

We also found variation in codon usage between rhodopsin secondary structures. Helices had a significantly higher proportion of codons recognized by abundant tRNAs compared to loops, a finding that implies there are local differences in the rate and accuracy of translation [17,34]. A handful of studies have linked tRNA abundances with codon usage in mammals [45,98-100], with rare codons associated with certain secondary structures such as turns, loops, beta strands, and domain boundaries [39,42,101,102]. Codons corresponding to less abundant tRNAs are thought to introduce pauses during translation, thereby enhancing correct folding (for review see [103]). For example, translational pausing is beneficial for the correct integration of yeast and plant transmembrane proteins into the endoplasmic reticulum [104,105]. For rhodopsin, not only are the transmembrane helical domains incorporated into the endoplasmic reticulum during elongation [106,107], but their proper alignment also depends on the attachment of properly folded intra-discal loop segments and the formation of a disulfide bond between cysteine side-chains at sites 110 and 187 [107,108]. As there are indications that protein folding can initiate in the ribosome exit tunnel [109], the use of slow codons in the loops could provide needed pauses during translation.

Alternatively, rhodopsin helices may simply experience tighter selection to minimize amino acid misincorporation, which can alter protein function or cause misfolding. However, we only found weak evidence for varying synonymous substitution rates between loops and helices, implying that selective differences between these regions are not strong. Substitution rates generally increased from the first- to the last-translated loop, suggesting that selective constraint on synonymous codons is weaker in the later loops. This may be because the protein is more robust to errors that cause folding disruptions when it is nearly fully folded. Rhodopsin helix residues contribute critically to the chemical environment of the chromophore binding pocket so slightly elevated selective constraint in these domains over the loops would be expected, but selection to pause translation in the loops by using rare codons cannot be ruled out.

### mRNA stability

We found a significantly higher proportion of GC-ending codons at paired sites versus non-paired sites in mRNA 2D structures. This suggests that the high GC-content at four-fold degenerate sites in mammalian rhodopsins may also be associated with maintaining mRNA stability. These nucleotides are thought to contribute more to mRNA stability because G:C pairs are more strongly bonded than A:T pairs [47,48] and they increase mRNA resistance to endo-ribonuclease activity, which cleave mRNAs at AU sites [46]. However, neither of these hypotheses explains the pervasive preference of C over G at four-fold degenerate sites in our dataset. Among mammals, there is a known exon-dependent preference for C over G at four-fold degenerate sites in the genomes of mice, rats [22], humans, and chimpanzees [110]. This was subsequently demonstrated to increase mRNA stability at four-fold degenerate sites; wild-type genes with the highest relative stability had a greater excess of C over G, and their stabilities decreased when C and G were swapped at four-fold degenerate sites [47]. However, our simulated G-C exchanges resulted in lower minimum free energy compared to the original sequences for all species. This suggests that, for our dataset, selection for mRNA stability may only be contributing to a general preference for GC-ending codons (not the specific preference for C-ending codons) in mammalian rhodopsin.

However, overly stable mRNA structures may also be a disadvantage given they can interfere with other processes such as spliceosome activity and translation initiation [111], and thus ultimately reduce translation speed. Selection for increased accuracy at conserved sites, increased translational speed, and for proper protein folding seem to take precedence over selection for mRNA stability in mammalian rhodopsin. Several other studies have reported conflicts in codon choice under multiple selection pressures. For example, Carlini et al. [112] showed that several highly transcribed genes avoided optimal codons that could generate adverse mRNA secondary structures in *Drosophila*, and Warnecke & Hurst [113] showed there was a trade-off between *Drosophila* translational efficiency and splicing regulation. The preference for G-ending codons in rhodopsin might also be the result of mutational bias; the proportion of G-ending codons among all four-fold codons was very similar to the G content in introns (26% on average in exons compared to 27% in introns). Any increases in mRNA stability that arise from G-ending codon bias may thus partly be a by-product of mutational bias. In addition, the significant GC-ending preference may partly be an artifact of the MFE algorithm’s tendency to minimize Gibbs energy by maximizing base-pairings. Resolved crystal structures will be necessary to confirm mRNA secondary structure in the future.

### Selection for splicing control at exonic splicing enhancer (ESE) regions

Research in humans has indicated that synonymous mutations can cause disease by disrupting splicing sites or ESE regions ([114]; for review see [6]). Studies that examine the evolution of splicing-associated regions, especially exon-intron splicing junctions and ESEs, have provided much insight on the selective constraint associated with splicing. For example, the human BRCA1 and CFTR genes have reduced synonymous substitution rates in regions containing an ESE (BRCA1: [115,116]; CFTR: [117]). More generally, a genome-wide human SNP study showed that SNP frequency was lower at synonymous sites in putative ESE hexamers than in non-ESE sequences [118]. An interspecies comparison of human, chimpanzee, and mouse orthologs also demonstrated that putative ESE regions showed significantly lower synonymous substitution rates than non-ESE regions [51]. Constraint on splicing enhancer regions in mammalian rhodopsins confirms another mechanism contributing to selection at synonymous sites. Given that our ESE analyses were limited to human and mouse, we suspect that a significant pattern may also become clearer with a larger species dataset.

## Conclusions

We found significant evidence for selection on synonymous sites in mammalian rhodopsin using phylogenetic likelihood models that explicitly differentiate between selection and mutational bias. These models indicated that within codon families, C-ending codons had the highest relative fitness. Furthermore, C-ending codons are associated with conserved residues and abundant cognate tRNAs, which suggests selection for increased translational accuracy and speed. Slightly elevated use of these codons in the helices over the loops, and slightly higher synonymous substitution rates in some loops, also suggest some influences from protein secondary structure. Additionally, synonymous site selection appears to contribute to mRNA stability and conservation of ESE regions. Our combined use of synonymous substitution models for detecting selection, and analytical approaches for detecting mechanistic effects on codon usage, demonstrate that post-transcriptional and translational processes are likely exerting selective constraint on the evolution of synonymous codons in mammalian rhodopsin. We expect that other highly expressed transmembrane proteins, such as others in the GPCR family, should display similar selection signals on synonymous codons. Our results highlight the importance of focusing attention on highly expressed genes in a broader phylogenetic context in order to better understand post-transcriptional and translational processes driving the evolution of synonymous substitutions.

## Competing interests

The authors declare they have no competing interests.

## Authors’ contributions

BSWC and JD designed the study. JD compiled the dataset, performed the initial analyses, constructed the figures and tables, and helped to draft the manuscript. SZD drafted the manuscript. AS contributed to design and implementation of statistical tests and helped to draft the manuscript. BSWC guided all aspects of the study, and helped to draft the manuscript. All authors read and approved the final manuscript.

## Supplementary Material

Additional file 1 **Table A1.** Accession numbers of resource records for all rhodopsin sequences downloaded from NCBI. **Table A2.** Nucleotide contents of four-fold degenerate codons and introns in mammalian rhodopsin genes. C4%, G4%, T4%, A4% represent the percentage of each nucleotide content within all four-fold degenerate codons while Ci%, Gi%, Ti%, Ai% represent those within introns. The introns here refer to all the introns in rhodopsin genes except the first intron, which contain regulatory regions and therefore may have more biased nucleotide content. **Table A3.** List of tRNA copy numbers for all the four-fold level degenerate codons in five mammalian species. For each amino acid and species, a single asterisk (*) indicates the tRNA species with the lowest gene copy number and a double asterisk (**) indicates the tRNA species with the highest gene copy number. The codons translated by these tRNAs (shown with arrows) were designated slow- and fast-translating respectively. Amino acids indicated with a triple asterisk (***) are six-fold degenerate, but we use only the four-fold sets (shown above) in our analyses (see Methods for details). **Table A4.** Codon fitness (F), usage bias (B), and cognate tRNA abundance (T) in five mammalian rhodopsins. **Table A5.** Free energy of mRNA secondary structure predicted by each rhodopsin coding sequence. MFE is minimum free energy. TE is thermodynamic ensemble. Click here for file

Additional file 2: Figure A1 Species cladogram for mammalian rhodopsins used in this study. Presented species relationships have been previously established in the literature [60-63]. Click here for file

Additional file 3: Figure A2 Synonymous substitution rates across sites of mammalian rhodopsin genes. The top boxes represent the eight helices in the 3D structure of rhodopsin associated with their positions in the gene. The main plot shows the variation of dS across sites, estimated under a distribution of three discrete categories in the Dual phylogenetic codon model of the _Hyphy_ package. The distribution of dS is drawn from codon 1 to codon 353, with regions in different exons highlighted with five different colors. Click here for file
