# Supplementary material for: Selection on synonymous codons in mammalian rhodopsins: a possible role in optimizing translational processes
Source: BMC Evol Biol. 2014 May 3;14:96. doi: 10.1186/1471-2148-14-96 (PMC4021273; doi:10.1186/1471-2148-14-96)
Supplement: Additional file 1 — Table A1. Accession numbers of resource records for all rhodopsin sequences downloaded from NCBI. Table A2. Nucleotide contents of four-fold degenerate codons and introns in mammalian rhodopsin genes. C4%, G4%, T4%, A4% represent the percentage of each nucleotide content within all four-fold degenerate codons while Ci%, Gi%, Ti%, Ai% represent those within introns. The introns here refer to all the introns in rhodopsin genes except the first intron, which contain regulatory regions and therefore may have more biased nucleotide content. Table A3. List of tRNA copy numbers for all the four-fold level degenerate codons in five mammalian species. For each amino acid and species, a single asterisk (*) indicates the tRNA species with the lowest gene copy number and a double asterisk (**) indicates the tRNA species with the highest gene copy number. The codons translated by these tRNAs (shown with arrows) were designated slow- and fast-translating respectively. Amino acids indicated with a triple asterisk (***) are six-fold degenerate, but we use only the four-fold sets (shown above) in our analyses (see Methods for details). Table A4. Codon fitness (F), usage bias (B), and cognate tRNA abundance (T) in five mammalian rhodopsins. Table A5. Free energy of mRNA secondary structure predicted by each rhodopsin coding sequence. MFE is minimum free energy. TE is thermodynamic ensemble. [file 1471-2148-14-96-S1.pdf]

Additional Table A1. Accession numbers of resource records for all rhodopsin sequences downloaded from NCBI.

Species common name	Scientific name	Accession number	Accession number (introns)	Codons	four-fold degenerate codons
Platypus	*Ornithorhynchus anatinus*	EF050076.1	EF050076.1	353	168
Fat-tailed dunnart	*Sminthopsis crassicaudata*	AY159786.2	N/A	348	162
white-eared opossum	*Caluromys philander*	AY313946.1	N/A	348	166
African elephant	*Loxodonta africana*	AY686752.1	N/A	348	172
West Indian Manatee	*Trichechus manatus*	AF055319.1	N/A	348	170
Cat	*Felis catus*	NM_001009242.1	AJ417432	348	174
Polar Bear	*Ursus maritimus*	AY883926.1	N/A	323	161
Dog	*Canis lupus familiaris*	NM_001008276.1	NC_006602.2	348	172
Cow	*Bos taurus*	NM_001014890.1	NC_007320.3	348	174
Pig	*Sus scrofa*	NM_214221.1	NC_010455.1	348	174
European Rabbit	*Oryctolagus cuniculus*	NM_001082349.1	N/A	348	171
Guinea Pig	*Cavia porcellus*	EF457995	N/A	348	168
Chinese Hamster	*Cricetulus griseus*	X61084.1	X61084.1	348	173
Mouse	*Mus musculus*	NM_145383	ENSMUST00000032471 (Ensemble database)	348	172
Galago	*Otolemur crassicaudatus*	AB112594.2	AB112594.2	348	174
Rhesus Macaque	*Macaca mulatta*	XM_001094250.1	ENSMMUT00000017859 (Ensemble database)	348	172
Human	*Homo sapiens*	NM_000539.2	ENST00000296271 (Ensemble database)	348	172

Additional Table A2. Nucleotide contents of four-fold degenerate codons and introns in mammalian rhodopsin genes.

Species	Exons				Introns*			
	C4%	G4%	T4%	A4%	Ci%	Gi%	Ti%	Ai%
Platypus	47	24	15	14	27	27	24	22
Echidna	43	27	19	11	26	26	25	23
Dunnart	46	16	20	18				
Opossum	46	12	27	15				
Elephant	53	28	10	9				
Manatee	54	25	8	13				
Cat	49	25	13	13	22	22	30	26
Polar bear	57	32	6	5				
Dog	52	24	11	13	28	26	24	22
Cow	55	32	9	4	29	29	21	21
Pig	53	25	10	12	29	28	24	19
Rabbit	54	33	8	5				
Guinea pig	56	21	14	9				
Hamster	49	24	13	14	26	27	23	24
Mouse	49	24	13	14	18	33	28	21
Galago	55	28	7	10	26	29	21	24
Macaque	55	27	10	8	29	26	22	23
Human	53	26	10	11	26	29	23	22

C4%, G4%, T4%, A4% represent the percentage of each nucleotide content within all four-fold degenerate codons while Ci%, Gi%, Ti%, Ai% represent those within introns. The introns here refer to all the introns in rhodopsin genes except the first intron, which contain regulatory regions and therefore may have more biased nucleotide content.

Additional Table A3. List of tRNA copy numbers for all the four-fold level degenerate codons in five mammalian species.

Amino Acid	Codon	tRNA	tRNA copy number						
			Human	Macaque	Mouse	Cow	Dog	Opossum	Platypus
**Ala**	*GC**T***	*AGC*	29**	15**	19**	30	14**	15**	83**
	*GC**C***	*GGC*	0	1	0	2	0	0	0
	*GC**G***	*CGC*	5*	5*	10*	17*	3*	6*	11*
	GCA	*TGC*	9	8	11	34**	11	9	17
**Gly**	*GG**T***	*ACC*	0	0	1	20	0	0	2
	*GG**C***	*GCC*	15**	11**	14**	62*	10	24**	40**
	*GG**G***	*CCC*	7*	7	7*	1315**	9*	8*	9*
	GGA	*TCC*	9	5*	7*	398	11**	11	15
**Pro**	*CC**T***	*AGG*	10**	8**	7	12**	8**	11**	19**
	*CC**C***	*GGG*	0	0	0	0	0	0	4
	*CC**G***	*CGG*	4*	4*	3*	5*	4*	3*	7*
	CCA	*TGG*	7	5	8**	9	8**	8	12
**Thr**	*AC**T***	*AGT*	10**	8**	9**	14**	8**	12**	23**
	*AC**C***	*GGT*	0	0	0	1	0	0	1
	*AC**G***	*CGT*	6*	4*	5	7*	5	3*	10*
	ACA	*TGT*	6*	6	4*	10	4*	7	18
**Val**	*GT**T***	*AAC*	11	6*	8	23	6	9**	23**
	*GT**C***	*GAC*	0	0	1	0	0	0	1
	*GT**G***	*CAC*	16**	12**	11**	45**	8**	9**	10*
	GTA	*TAC*	5*	7	3*	22*	5*	5*	15
**Ser*** **	*TC**T***	*AGA*	11**	9**	9**	14	10**	12**	16**
	*TC**C***	*GGA*	0	0	1	30**	0	0	4
	*TC**G***	*CGA*	4*	4*	3*	7	7	3*	8
	TCA	*TGA*	5	5	3*	6*	5*	6	6*
**Arg*** **	*CG**T***	*ACG*	7**	7**	6**	14**	6	5**	12
	*CG**C***	*GCG*	0	0	0	9	0	0	0
	*CG**G***	*CCG*	4*	4*	3*	14**	4*	4*	14**
	CGA	*TCG*	6	5	5	12*	15**	5**	7*
**Leu*** **	*CT**T***	*AAG*	12**	9**	8	11**	4*	8	8
	*CT**C***	*GAG*	0	0	0	0	0	0	0
	*CT**G***	*CAG*	10	5	10**	9	7**	11**	18**
	CTA	*TAG*	3*	4*	3*	5*	4*	6*	7*

For each amino acid and species, a single asterisk (*) indicates the tRNA species with the lowest gene copy number and a double asterisk (**) indicates the tRNA species with the highest gene copy number. The codons translated by these tRNAs (shown with arrows) were designated slow- and fast-translating respectively. Amino acids indicated with a triple asterisk (***) are six-fold degenerate, but we use only the four-fold sets (shown above) in our analyses (see methods for details).

Table A3 continued.

Amino Acid	Codon	tRNA	tRNA copy number Hamster	Cat	Rabbit	Pig	Guinea pig	Elephant
**Ala**	*GCT*	*AGC*	22**	18**	21**	24**	28**	54**
	GCC	*GGC*	1	1	0	0	4	0
	GCG	*CGC*	6*	6*	4*	7*	5*	15*
	GCA	*TGC*	15	8	9	17	9	53
**Gly**	*GGT*	*ACC*	1	1	0	0	0	4
	GGC	*GCC*	14**	9	21**	16**	11**	33*
	GGG	*CCC*	7	6*	16	7*	6*	72
	GGA	*TCC*	6*	40**	14*	7*	6*	619**
**Pro**	*CCT*	*AGG*	7**	7*	9**	8**	8**	16**
	CCC	*GGG*	0	1	0	0	0	0
	CCG	*CGG*	3*	7*	4*	2*	3*	3*
	CCA	*TGG*	6	52**	5	4	4	14
**Thr**	*ACT*	*AGT*	8**	7	8**	8**	10**	14
	ACC	*GGT*	1	0	1	0	0	1
	ACG	*CGT*	4*	5*	5*	5*	3*	10*
	ACA	*TGT*	4*	11**	7	8**	4	22**
**Val**	*GTT*	*AAC*	13**	8**	12	8*	6**	41
	GTC	*GAC*	0	0	0	0	1	1
	GTG	*CAC*	9	6	14**	9**	6**	35*
	GTA	*TAC*	5*	5*	8*	9**	3*	50**
**Ser*** **	*TCT*	*AGA*	6**	7	10**	13**	8**	29**
	TCC	*GGA*	0	1	0	0	0	0
	TCG	*CGA*	3*	5*	5	4*	4	4*
	TCA	*TGA*	3*	11**	4*	7	3*	7
**Arg*** **	*CGT*	*ACG*	6**	7*	7**	4	7**	14
	CGC	*GCG*	0	6*	0	0	0	0
	CGG	*CCG*	3*	23	4*	3*	3*	3*
	CGA	*TCG*	6**	985**	5	6**	4	18**
**Leu*** **	*CTT*	*AAG*	5	5	5	6**	5	22**
	CTC	*GAG*	0	0	0	0	0	0
	CTG	*CAG*	10**	1*	16**	3*	6**	10*
	CTA	*TAG*	4*	26**	4*	3*	3*	11

Additional Table A4. Codon fitness (F), usage bias (B), and cognate tRNA abundance (T) in five mammalian rhodopsins.

Amino acid	Codon	F	Human		Macaque		Mouse		Cow		Dog	
			B	T	B	T	B	T	B	T	B	T
**Ala**	GCY(T)			+		+		+				+
	**GCY(C)**	+	+	+	+	+	+	+	+		+	+
	GCG											
	GCA									+		
**Gly**	GGY(T)			+		+		+				
	**GGY(C)**	+	+	+	+	+	+	+	+		+	
	GGG									+		
	GGA											+
**Pro**	CCY(T)			+		+				+		+
	**CCY(C)**	+	+	+	+	+	+		+	+	+	+
	CCG											
	CCA							+				
**Thr**	ACY(T)			+		+		+		+		+
	**ACY(C)**	+	+	+	+	+	+	+	+	+	+	+
	ACG											
	ACA											
**Val**	GTY(T)											
	**GTY(C)**	+	+		+		+		+		+	
	GTG			+		+		+		+		+
	GTA											
**Ser***	TCY(T)			+		+		+		+		+
	**TCY(C)**	+	+	+	+	+	+	+	+	+	+	+
	TCG											
	TCA											
**Arg***	CGY(T)			+		+		+		+		
	**CGY(C)**	+	+	+	+	+	+	+	+	+	+	
	CGG											
	CGA											+
**Leu***	CTY(T)			+		+				+		
	CTY(C)			+		+				+		
	**CTG**	+	+		+		+	+	+		+	+
	CTA											

Asterisks (*) indicate amino acids that are six-fold degenerate (only four-fold codons shown). Plus signs (+) indicate the codon with the highest fitness, most usage bias, and that corresponds with the most abundant cognate tRNAs. Shading highlights, for each amino acid, the codon with the highest fitness, most biased usage and most abundant cognate tRNAs.

Additional Table A5. Free energy of mRNA secondary structure predicted by each rhodopsin coding sequence.

Species	MFE ΔG (kcal/mol)		TE ΔG (kcal/mol)	
	Original	GC swapped	Original	GC swapped
Platypus	-365.60	-404.54	-385.07	-424.58
Echidna	-371.48	-383.64	-392.09	-405.35
Dunnart	-356.80	-372.80	-375.04	-393.87
Opossum	-341.52	-374.19	-360.99	-393.75
Elephant	-393.87	-420.25	-411.88	-440.54
Manatee	-394.66	-435.20	-412.95	-454.36
Cat	-384.82	-416.29	-400.85	-432.98
Dog	-409.08	-425.20	-423.66	-444.15
Cow	-411.00	-439.14	-429.87	-457.39
Pig	-389.74	-422.97	-408.47	-438.64
Rabbit	-426.55	-437.00	-441.50	-461.77
Guinea Pig	-380.75	-415.02	-397.79	-435.00
Hamster	-380.89	-399.61	-396.95	-416.26
Mouse	-382.76	-416.94	-400.66	-436.72
Galago	-405.17	-415.6	-423.39	-435.06
Macaque	-398.35	-440.10	-416.79	-456.84
Human	-409.08	-441.23	-423.66	-461.51

MFE is minimum free energy. TE is thermodynamic ensemble.
