# Supplementary figures and images for: Selection on synonymous codons in mammalian rhodopsins: a possible role in optimizing translational processes
Source: BMC Evol Biol. 2014 May 3;14:96. doi: 10.1186/1471-2148-14-96 (PMC4021273; doi:10.1186/1471-2148-14-96)

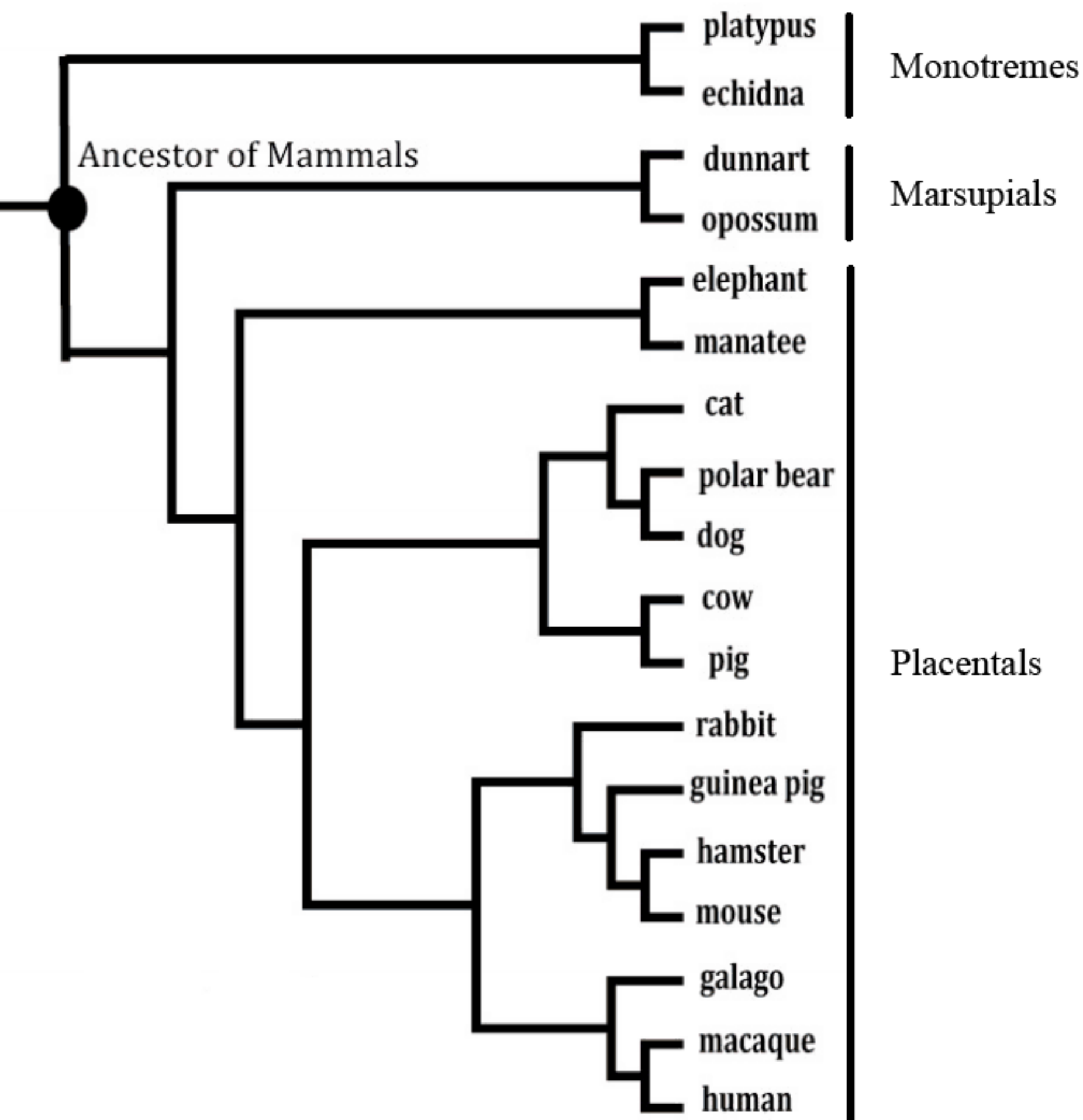

Supplement: Additional file 2: Figure A1 — Species cladogram for mammalian rhodopsins used in this study. Presented species relationships have been previously established in the literature [60-63]. [file 1471-2148-14-96-S2.pdf]

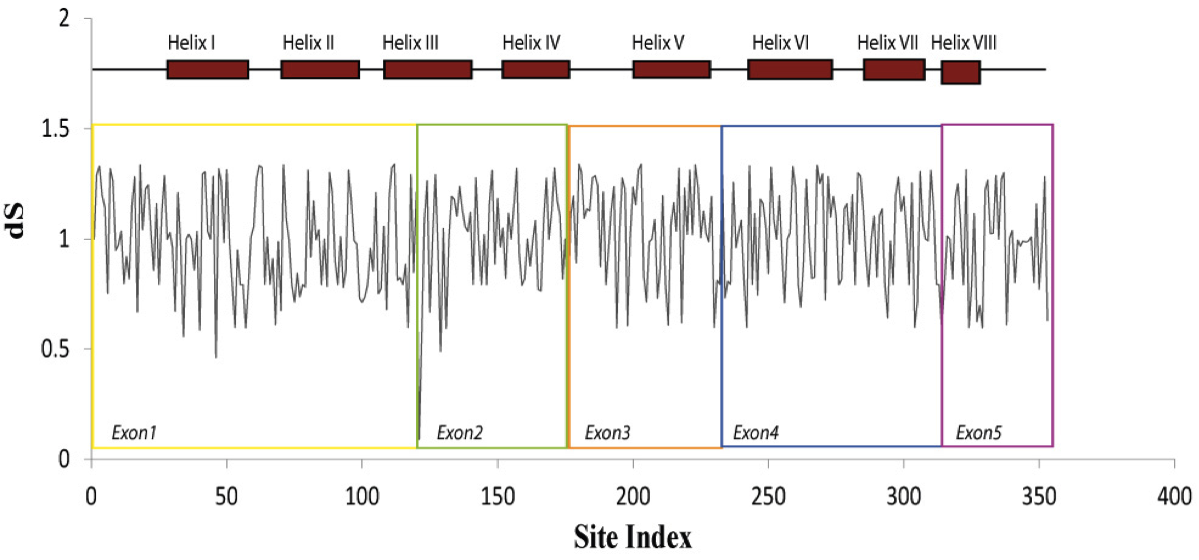

Supplement: Additional file 3: Figure A2 — Synonymous substitution rates across sites of mammalian rhodopsin genes. The top boxes represent the eight helices in the 3D structure of rhodopsin associated with their positions in the gene. The main plot shows the variation of dS across sites, estimated under a distribution of three discrete categories in the Dual phylogenetic codon model of the Hyphy package. The distribution of dS is drawn from codon 1 to codon 353, with regions in different exons highlighted with five different colors. [file 1471-2148-14-96-S3.png]
